# Survey on Nutrition in Neurological Intensive Care Units (SONNIC)—A Cross-Sectional Survey among German-Speaking Neurointensivists on Medical Nutritional Therapy

**DOI:** 10.3390/jcm13020447

**Published:** 2024-01-13

**Authors:** Leon Gehri, Moritz L. Schmidbauer, Timon Putz, Luka Ratkovic, Andreas Maskos, Cedric Zeisberger, Julia Zibold, Konstantinos Dimitriadis, on behalf of the IGNITE Study Group

**Affiliations:** Department of Neurology, LMU University Hospital, Ludwig-Maximilians-University Munich, 81377 Munich, Germany; leon.gehri@med.uni-muenchen.de (L.G.); moritz.schmidbauer@med.uni-muenchen.de (M.L.S.); luka.ratkovic@med.uni-muenchen.de (L.R.); andreas.maskos@med.uni-muenchen.de (A.M.); cedric.zeisberger@med.uni-muenchen.de (C.Z.); julia.zibold@med.uni-muenchen.de (J.Z.)

**Keywords:** nutrition, intensive care unit, survey, medical nutritional therapy, nutritional management, nutritional practices, nutritional concepts, neurointensive care unit, NICU, ICU

## Abstract

Medical nutritional therapy (MNT) in neurointensive care units (NICUs) is both particularly relevant and challenging due to prolonged analgosedation, immobilization, disorders of consciousness, and the high prevalence of dysphagia. Moreover, current guideline recommendations predominantly address the general intensive care unit (ICU) population, overlooking specific characteristics of neurological patients. We, therefore, conducted a web-based, cross-sectional survey for German-speaking neurointensivists mapping the clinical practices of MNT on NICUs to identify research gaps and common grounds for future clinical trials. A total of 25.9% (56/216) NICU representatives responded to our questionnaire. A total of 78.2% (43/55) were neurologist and 63% (34/54) held a leadership role. Overall, 80.4% (41/51) had established a standard operating procedure (SOP), largely based on the DGEM-Guideline (53.7%; 22/41), followed by the ESPEN-Guideline (14.6%; 6/41). Upon admission, 36% (18/50) conducted a risk stratification, with 83.3% primarily relying on past medical history (15/18) and clinical gestalt (15/18). Energy expenditure (EE) was measured or calculated by 75% (36/48), with 72.2% (26/36) using pragmatic weight-based equations. Indirect calorimetry was used by 19.4% (7/36). A total of 83.3% (30/36) used the patient’s serum glucose level as the primary biomarker to monitor metabolic tolerance. SOPs regarding ICU-Acquired Weakness (ICUAW) were found in 8.9% (4/45) of respondents. Overall, guideline adherence was 47%. In summary, this is, to the best of our knowledge, the first study systematically describing the currently applied concepts of MNT on NICUs. The data reveal great variations in the implementation of guideline recommendations, indicating the need for further research and tailored approaches to optimize nutritional therapy in neurointensive care settings.

## 1. Introduction

Malnutrition, encompassing both under- and over-nourishment, has been linked to adverse outcomes in intensive care, such as prolonged ventilation and hospitalization, increased susceptibility to infections and higher mortality [1,2]. Nowadays, the ubiquity and significance of medical nutritional therapy (MNT) on intensive care units (ICUs) is acknowledged and has been incorporated into established international guidelines published by the American Society for Parenteral and Enteral Nutrition (ASPEN), the European Society of Intensive Care Medicine (ESICM), the European Society for Clinical Nutrition and Metabolism (ESPEN), and the guideline of the German Society for Nutritional Medicine (DGEM) [3,4,5,6,7].

As patients in neurointensive care, compared to the general ICU population, receive prolonged analgosedation, are immobilized due to motor deficits, and have a high incidence of dysphagia and disorders of consciousness, addressing malnutrition via medical nutritional therapy (MNT) is of paramount importance [8,9,10,11]. Yet, existing guidelines on MNT in intensive care are primarily tailored for the general ICU population [1,2,3,4], illustrating the scarce evidence for NICU patients. Moreover, some guideline recommendations are based on expert consensus and some recommendations, like the implementation of indirect calorimetry, spark debates on feasibility and clinical value. While the implementation of such MNT concepts has been investigated to some extent on general ICUs, ref. [12] data focusing on the characteristics of NICUs are absent. Overall, this lack of evidence and the missing specific recommendations leaves a significant knowledge gap regarding MNT for NICU patients.

Thus, we hypothesize that MNT in neurocritical care is heterogenous and exhibits a poor adherence to existing guidelines. Accordingly, this study aims to comprehensively map the clinical practices of MNT on NICUs across Germany to identify research gaps and common grounds for future clinical trials.

## 2. Materials and Methods

### 2.1. Survey Design and Distribution

To provide an overview of MNT and its implementation on neurological and neurosurgical intensive care units across Germany, a cross-sectional study was designed. The German Society for Neurointensive and Emergency Care (DGNI) has registered 235 NICUS in Germany on their website (https://www.dgni.de/verzeichnis-neurointensivstationen.html, accessed on 15 January 2023). We took the initiative to update this register by verifying email addresses and, if necessary, reached out to the respective centers to ask for updated contact information. The register was then used as the target population and sample frame. Subsequently, a total of 231 ICUs received an invitation link via email. In cases where physicians were not identifiable, the link was sent to the head of the department. Fifteen invitations were undeliverable. The survey responses were collected between 6 May 2023, and 18 June 2023. A reminder was sent three weeks after the initial invitation. This study received approval from the institutional ethics committee of Ludwig-Maximilians-University Munich, and the requirement for written consent was waived on the 20 December 2022 (project number: 22-1123KB).

### 2.2. Questionnaire

The survey was constructed using the web-based platform Research Electronic Data Capture (RedCap, Version 13.4.12, Vanderbilt University, Nashville, TN, USA). Using the “think-aloud” method [13,14], the questionnaire underwent multiple reviews, discussions and pretesting within the group of authors as well as external nutrition experts. The final survey consisted of 139 questions (72× single-choice; 35× multiple-choice, and 32× open-label questions). Depending on the answers given by the respondent, non-applicable questions were hidden. The questions covered demographics, quality management, nutritional risk assessment, calculation and monitoring of energy expenditure, composition and monitoring of nutritional therapy, monitoring of ICU-Aquired Weakness (ICUAW), and mobilization strategies. The median processing time to complete the survey was 13 min and 40 s. Neither patient data nor the respondent’s identity were collected. To ensure consistent interpretation and minimize question-skipping, closed-ended questions and popup questions were predominantly used in our survey. Rating scales included five to seven alignments labeled with clear and unambiguous words. Answers were randomized and rotated to address primacy and recency effects. Examples were included to avoid lack of clarity.

### 2.3. Data Analysis

Data analysis was performed using Microsoft Excel (Version 16.67, Redmond, DC, USA) and Graph Pad Prism (Version 10.0.3, San Diego, CA, USA). Descriptive statistics are reported as frequencies and means with standard deviation (SD) for continuous data. Multiprofessional treatment teams (MTT) were defined as a minimum of three professions including physicians, nurses, and nutrition experts. Standard treatment teams (STT) were defined as a team consisting of only physicians and nurses. To elucidate potential differences in management according to demographic characteristics, a comparison of guideline adherence was conducted among the following groups: type of institution (academic hospital vs. non-academic), subspeciality (neurology vs. others), leadership position on ICU (yes vs. no), years of experience in ICU care (>5 years vs. <5 years), annual number of patients (>450 vs. <450), structure of ICU at the institution (neurology led vs. others), and the type of treatment teams (MTT vs. STT). The overall adherence represents the mean of the adherence of the subcategories. Chi-squared test with Yates’s correction was used in the analysis of contingency tables. A *p*-value ≤ 0.05 was considered statistically significant.

## 3. Results

### 3.1. Demographics of Participating Neurointensivists and Corresponding ICUs

Table 1 displays the characteristics of the respondents to our survey and their corresponding ICUs. Out of 216 delivered invitations, a total of 56 ICU representatives participated, representing a respondence rate of 25.9%. The mean age of the participating neurointensivists was 46 years (SD 9.2). A total of 78.2% (43/55) were neurologists, and 10.9% were bord-certified anesthesiologists (6/55). A majority had extensive experience in intensive care medicine (66.7% (36/54) with >5 years of experience). Furthermore, 63% (34/54) of the respondents held leadership positions within their units. Around half of respondents practiced at an academic institution (43.6% (24/55)).

### 3.2. Standardization and Multidisciplinarity of MNT

**Figure 1:**
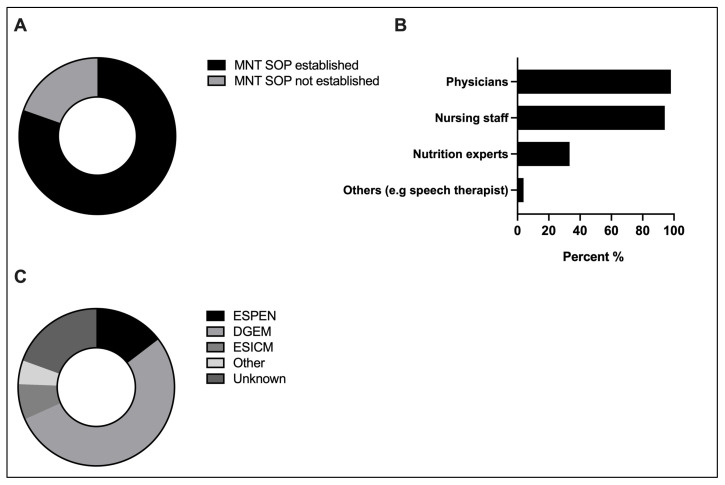
SOPs and multidisciplinarity in MNT: (**A**) Proportion of centers with SOP for MNT (*n* = 51). (**B**) Relevant guidelines forming MNT SOPs (*n* = 41). (**C**) Professions involved in MNT (*n* = 51, multiple selection). SOP, standard operating procedure. MNT, medical nutritional therapy. ESPEN, European Society for Clinical Nutrition and Metabolism. DGEM, German Society for Nutritional Medicine. ESICM, European Society of Intensive Care Medicine.

A total of 80.4% (41/51) of respondents were confirmed to have implemented a SOP for managing MNT. The remaining 10 NICUs used various approaches: 60% (6/10) relied on a combination of clinical expertise and guidelines, 30% (3/10) based their approach predominantly on clinical expertise, and 10% (1/10) on guidelines. A total of 53.7% (22/41) based their SOP on the DGEM guideline, followed by 14.6% (6/41) on the ESPEN guideline, and 7.3% (3/41) on the ESICM guideline. A total of 19.5% (8/41) were uncertain about the specific guideline their SOP was derived from. MNT was managed by different professions, yet most centers had teams consisting of physicians (multiple selection, 98.0% (50/51)) and nursing staff (multiple selection, 94.1% (48/51)). A total of 66% (33/50) stated not to believe that their nutritional concepts are uniformly implemented by all professional groups involved (Figure 1).

### 3.3. Assessment of Nutritional Status

**Figure 2:**
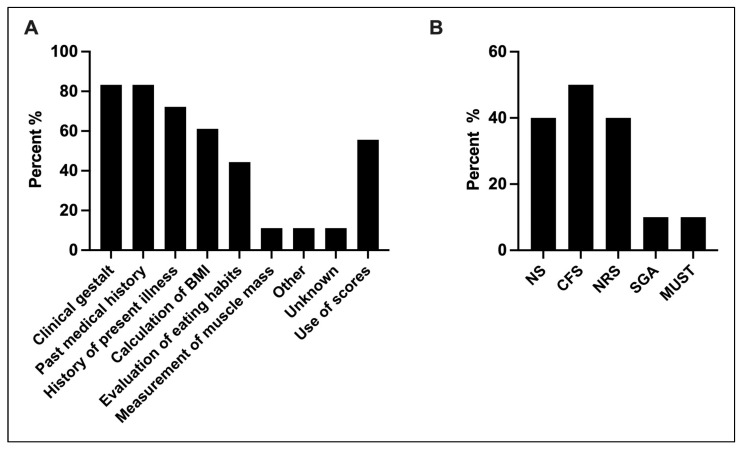
Methods and scores used for nutritional risk assessment: (**A**) Methods used for nutritional risk assessment at admission (*n* = 18, multiple selection). (**B**) Use of scores for nutritional risk assessment (*n* = 10, multiple selection). NS, nutric score. CFS, Clinical Frailty Score. NRS, Nutritional Risk Screening. SGA, Subjective Global Assessment. MUST, Malnutrition Universal Screening Tool.

Overall, 36% (18/50) screened patients to identify those with high risk for malnutrition. Among those, 83.3% (15/18) used past medical history and clinical gestalt as primary methods for risk stratification. Specific risk stratification scores were used by 55.6% (10/18; Clinical Frailty Score: 50% (5/10), Nutric Score and Nutritional Risk Screening: 40% (4/10)) (Figure 2). Each patient’s individual body height at admission was determined by 70% (35/50) (mostly estimated by health care workers). Only 40% (14/35) utilized a tape measure. Similarly, 70.3% (26/37) estimated patient body weight, followed by 48.6% (18/37) that took this information from third-party sources. Beds with a weight scale option were used by 37.8% (14/37), and mechanical bed scales were used by 24.3% (9/37).

### 3.4. Determination of Energy Expenditure

In total, 75% (36/48) claimed to either measure or calculate patient-specific energy expenditure (EE). Within this group, 72.2% (26/36) used pragmatic body-weight-based equations, with 24 kcal/kilogram of body weight (kgBw)/day (50% (18/36)) and 25 kcal/kg body weight/day (16.67% (6/36)) being the most common. Other predictive equations were used by 33.3% (12/36), primarily the Harris–Benedict equation (22.2% (8/36)). Indirect calorimetry on the other hand was used by 19.4% (7/36). Yet, 28.6% (2/7) uses it for research purposes and not in clinical routines. Lastly, rough estimates for EE were chosen by 13.9% (5/36) of participating physicians.

**Figure 3:**
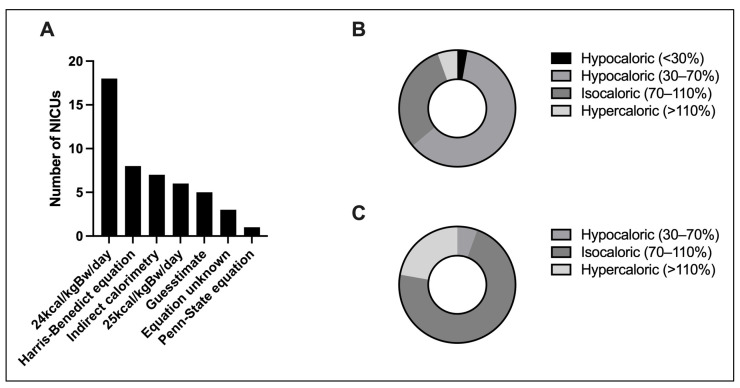
Determination of energy expenditure: (**A**) Methods used to determine EE (*n* = 36, multiple selection). (**B**) Caloric targets relative to calculated EE in the acute phase (d 0–2, *n* = 36). (**C**) Caloric targets relative to calculated EE in the post-acute phase (d 3–7, *n* = 36). Kcal/kgBw/day, kilocalories per kilogram bodyweight per day. EE, energy expenditure. d, day.

A total of 83.7% (36/43) differentiated the caloric target based on the phases of critical illness. For the acute phase (0–48 h), 61.1% (22/36) administered a hypocaloric target (30–70%) of the measured or calculated EE; an isocaloric target in the post-acute phase (3–7 d) was aimed at by 72.2% (26/36) (Figure 3).

### 3.5. Protein Targets

**Figure 4:**
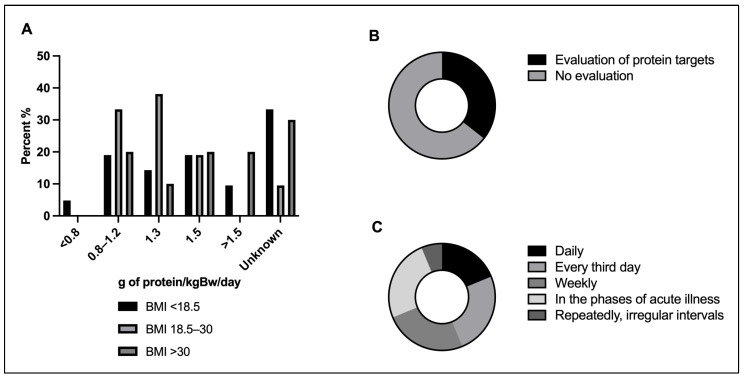
Protein target and evaluation: (**A**) Protein targets relative to BMI (*n* = 21). (**B**) Proportion of centers setting protein targets (*n* = 45). (**C**) Frequency of protein target evaluation (*n* = 16). BMI, Body Mass Index. g of protein/kgBw/day, grams of protein per kilogram bodyweight per day.

A total of 81.3% (39/48) of all clinicians individualized nutrient supply. Of those, 56.4% (22/39) expressed agreement to set a target value for protein intake for non-obese/non-cachectic patients. A target of 1.3 g/kgBw/day was most common and endorsed by 38.1% (8/21), followed by a range of 0.8–1.2 g/kgBw/day favored by 33.3% (7/21), and 1.5 g/kgBw/day supported by 19.0% (4/21). Notably, an ongoing evaluation of protein targets throughout the stay, whether based on body weight or the target value itself, was implemented in 35.6% (16/45) of the participating physicians (Figure 4).

### 3.6. Monitoring of Metabolic Tolerance and Energy Expenditure

**Figure 5:**
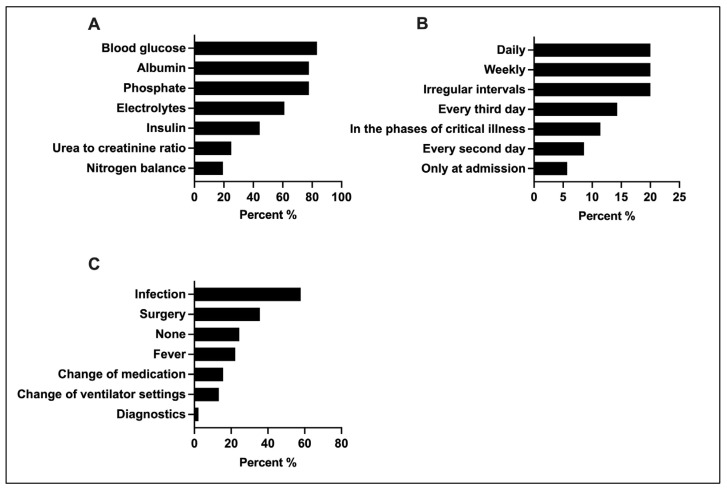
Monitoring of metabolic tolerance and EE: (**A**) Blood- and urine-based biomarkers used for monitoring metabolic tolerance (*n* = 36, multiple selection). (**B**) Frequency of metabolic tolerance monitoring (*n* = 35). (**C**) Clinical scenarios promoting re-evaluation of EE (*n* = 45; multiple selection). EE, energy expenditure.

Most clinicians monitored blood- and urine-based biomarkers (80% (36/45)). Of those, 83.3% (30/36) used the serum glucose level as the primary biomarker to monitor metabolic tolerance, followed by serum phosphate and albumin (77.8% (28/36)). The intervals used to monitor metabolic tolerance varied between regular daily or weekly intervals to irregular controls (equal distribution, 20% (7/35)). Re-evaluations of EE were mostly triggered by changes of patient’s medical condition (mostly new infections, 57.8% (26/45), or new surgical interventions, 35.6% (16/45)) (Figure 5).

### 3.7. ICU-Acquired Weakness

**Figure 6:**
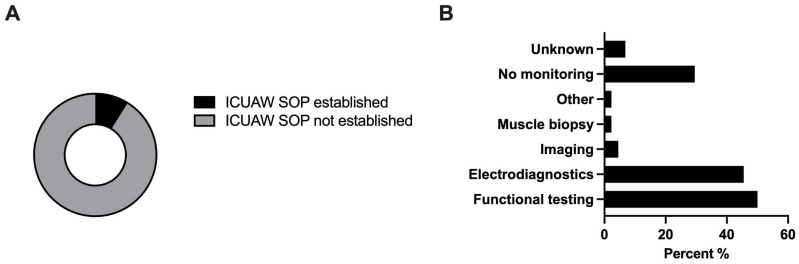
Management of ICUAW: (**A**) Proportion of centers with established ICUAW SOPs (*n* = 45). (**B**) ICUAW monitoring (*n* = 44, multiple selection). SOP, standard operating procedure. *n*, number.

The majority (91.1% (41/45)) had not implemented a SOP regarding the management of ICUAW so far. Still, 70% (31/44) screened for ICUAW, mostly via functional testing (50% (22/44)). Among those, physical examination was used by 95.5% (21/22), hand grip tests by 31.8% (7/22), and walking tests by 13.6% (3/22). In functional testing, 45.5% (20/44) applied electrodiagnostic methods (nerve conduction studies 90% (18/20); electromyography 75% (15/20)) (Figure 6).

### 3.8. Guideline Adherence

Overall, adherence of local practices to DGEM/ESPEN guidelines was 47% (Table 2 and Table S2). No significant differences in guideline adherence were found in any of the prespecified subgroups (Supplemental Table S1).

## 4. Discussion

This cross-sectional survey systematically assessed prevailing nutritional concepts within neurointensive care units. The key findings are as follows: (i) MNT is standardized via SOP and routinely managed by physicians and nursing staff in most centers; (ii) Stratification of risk for malnutrition upon admission to the NICU is not well established in clinical practice; (iii) EE is predominantly computed using pragmatic weight-based equations—a minority of NICUs employ indirect calorimetry; (iv) Nutrient supply, particularly protein targets and monitoring of metabolic tolerance, are part of good clinical practice; (v) However, the overall adherence of local practices to DGEM/ESPEN guidelines is low.

Previously, standardization of MNT in non-NICU patients resulted in a higher caloric intake and better functional outcome [15,16]. The ACCEPT study observed a significant reduction in length of hospitalization and showed a trend towards reduced mortality and a significant increase in the duration of enteral nutrition [15]. Barr et al. detected a shortened duration of mechanical ventilation [16]. Accordingly, the DGEM-Guideline advocates for a feeding protocol in clinical practice with a strong consensus (100%). In our findings, however, 20% of respondents had not implemented a SOP yet. In a recent similar survey conducted among general ICUs in Italy, almost half of all responders did not use structured nutritional protocols [12].

Similarly, risk assessment for malnutrition was not routinely applied in German NICUs, although an association between nutrition status upon ICU admission and mortality has been described previously [17,18]. Mogensen et al. noted an association between nutrition status and mortality in a sizable cohort of 6518 medical or surgical ICU patients [17]. Moisey et al. substantiated this finding in his observational study, which included 149 severely injured elderly patients, where the mortality rate among patients with preexisting sarcopenia was more than twice as high compared to patients who were not sarcopenic. Moreover, a decreased muscle index was significantly associated with a higher mortality rate (OR = 0.93; 95% CI 0.875 −0.997; *p* = 0.025) [18]. Although similar studies in NICU patients are scarce, we believe pre-existing malnutrition might be of even more importance in this cohort as the prevalence of disorders of consciousness, dysphagia, prolonged analgosedation, immobilization, and cognitive disorders typically complicate MNT [8,9,10,11,19]. Therefore, an assessment of nutrition status upon NICUs admission is important to identify patients at risk and consequently personalize nutritional therapy. This recommendation also found strong consensus in DGEM- and ESPEN-guidelines [4,6].

For the assessment of general ICU patients, the DGEM-guideline suggests that the subjective global assessment (SGA) may be used to assess nutrition status [6]. The ESPEN-guideline mentions the Nutritional Risk Screening (NRS) but does not endorse it due to lack of strong evidence [4]. However, DGEM explicitly advises against using the NRS score due to its allocation of three points to severe illness. This leads to an undifferentiated high-risk categorization for malnutrition in almost all ICU patients and especially applies to NICU patients with acute brain injury [6]. In a trial by Zhang et al. among NICU patients, 87.1% were classified as high risk according to the NRS score, while only 15.7% and 28.6% were categorized this way by the NUTRIC and mNUTRIC score, respectively. Moreover, the 28-day mortality prediction of both NUTRIC and mNUTRIC scores were significantly more accurate compared to the NRS score in this NICU cohort [20]. While similar studies in non-NICU cohorts have also demonstrated significant differences in predictive values in favor of NUTRIC and mNUTRIC scores, the number of false positive NRS scores of NICU patients is much higher [20,21]. However, it is important to note that this study is limited to a single-center observation, emphasizing the need for further research to identify an adequate tool for the NICU setting. Meanwhile, we recommend a routine screening of NICU patients on admission. As for the screening tool, the use of the SGA (as proposed by the DGEM guideline), NUTRIC or mNUTRIC score, or a combination of both (SGA and NUTRIC) seems to be reasonable.

Energy expenditure serves as an important reference for metabolic monitoring and the determination of exogenous substrate intake. The use of indirect calorimetry to guide MNT and improve clinical outcomes remains a topic of controversy, and is often not reported or evaluated in the NICU setting [22,23,24,25]. The TICACOS international multicenter study by Singer et al. failed to reach its primary endpoint but found a tendency towards lower mortality and decreased infection rates in the group undergoing IC-guided MNT compared to a control group with predictive equations as the basis for MNT [24]. Nonetheless, national and international guidelines and consensus statements promote the daily use of indirect calorimetry [3,4,5,6,26]. Strikingly, only seven out of the surveyed NICUs employed this method, indicating a substantial gap between recommended and clinical practice. This result was in line with an Italian survey on general ICU patients [12]. It is important to recognize that, while indirect calorimetry is a highly precise approach and is considered the gold standard, its implementation requires substantial financial and human resources; also, it exhibits technical limitations and might, therefore, not be applicable in a broader context [27]. Guidelines further emphasize that, if indirect calorimetry is not accessible, alternative methods like predictive and pragmatic body-weight-based equations should be employed. Although the level of evidence is low, predictive equations have been found to have low accuracy rates and a high degree of variability compared to IC in intensive care settings [28,29,30,31,32,33]. Even if the equations were a near perfect approximation of indirect calorimetry, they heavily rely on accurate measurements of patients’ weight and height. As these parameters can be either estimated, measured directly, or obtained from reliable sources like relatives, fluctuations in the respective results are to be expected [27]. Moreover, most equations do not include variables that change over the course of the disease and ICU stay, which makes their validity questionable. The results of our survey, which indicate that most centers relied on estimations, highlight the need for a reassessment of the methods employed to determine body weight and height. Beds equipped with weight-measuring capabilities or mechanical bed scales offer an appropriate alternative to estimation, leading to greater precision in the application of predictive equations. 

The survey also revealed heterogeneity in the setting of protein targets. While guidelines do specify concrete target values, well-defined targets, timing, and the effect of a high protein intake on outcomes are subject to recent debates [34,35,36]. The latest multicenter trial by Heyland et al. (EFFORT trial) included 1301 critically ill patients and observed no differences in 60-day survival among the two groups of high-dose (≥2.2 g/kg per day) and regular-dose protein (≤1.2 g/kg per day) (hazard ratio 0.91; 95% CI 0.77–1.07; *p* = 0.27). Yet, in a subgroup with acute kidney injury, higher protein provision even proved to be harmful [34]. DGEM defines a protein target of 1.0 g or 1.2 g/kg of actual body weight per day and ESPEN recommends a target of 1.3 g/kg protein equivalents per day during critical illness [4,6]. Trials on NICU patients accounting for their distinct patient characteristics, like preexisting malnutrition, neuromuscular injury, or specific disease (for example, acute brain injury vs. acute inflammatory demyelinating polyneuropathy), are pivotal to define specific targets for this population.

Monitoring of metabolic tolerance is also part of good clinical practice and was most recently included in the updated ESPEN guideline and position paper of the German Interdisciplinary Association for Intensive and Emergency Medicine [5,26]. While caloric intake should generally not exceed the measured REE, metabolic monitoring also accounts for endogenous substrate supply during the catabolic state. Consequently, the actual calories provided may vary based on the monitoring of metabolic tolerance like blood glucose and phosphate levels [26]. Although trials comparing outcomes using metabolic monitoring versus standards of care are lacking; the concept is convincing on a pathophysiologic level [37,38,39]. The lack of evidence for NICU patients is reflected by the heterogeneity in metabolic monitoring strategies in our survey. Results of this survey could help identify a common ground for the design of clinical trial protocols.

As inadequate nourishment and lack of mobilization, both common factors found in NICU cohorts, emerge as risk factors for ICUAW, its standardized assessment of NICU is of supreme importance [40]. Yet, less than 10% of respondents declared to have established a SOP regarding ICUAW. However, 70% screened for ICUAW, mainly via physical examination. This discrepancy may be explained by the fact that most of our respondents are neurologists and routinely perform clinical examinations, thereby screening for flaccid tetraparesis and other hallmarks of ICUAW. However, as physical examination is not very sensitive in the context of NICU patients unable to follow commands, we propose to introduce SOPs of ICUAW to standardize diagnoses and treatment.

Future investigations should particularly direct attention towards the identified re-search gaps. First, screening for patients with high risk of malnutrition exhibits great heterogeneity. Accordingly, there is a need to identify and validate a suitable screening tool that specifically suits NICU patients. These efforts should be paralleled by investigations providing evidence for better patient outcomes, stratifying MNT according to the risk of malnutrition. Second, IC in the context of neurocritical care needs to prove its superiority over conventional formulas to be ready for prime time. Third, the impact of nutrition experts as part of the neurocritical care team on patient outcomes needs to be further investigated [41,42]. Finally, there is a necessity to establish a consensus in the design of protocols for MNT trials that respect the pathophysiology of critically-ill patients with acute nervous system disorders to enable better planning of prospective interventional studies in the future.

Although our survey was performed only among German neurocritical care physicians, our results are in line with a similar study in a general ICU population in Italy [12], thereby suggesting external validity. Furthermore, trends identified in our study might be relevant for NICUs in other regions as well, since population characteristics, treatment strategies, and infrastructure, at least in other high-income countries, have many similarities. Consequently, this study serves as an example for similar healthcare facilities worldwide to conduct comparable assessments, prompting a critical re-evaluation of their respective management of MNT practices.

Despite achieving a response rate of 26%, the participating neurointensivists may not comprehensively represent the entirety of nutritional approaches and concepts and consecutive reporting bias may be present. Although we undertook peer-reviewing and pretesting efforts, response bias is of concern. Likewise, and inherent to questionnaires, social desirability response set may be an issue. Moreover, not all survey respondents provided comprehensive answers, resulting in missing data that are likely to have impacted the validity of our findings. The strengths of our survey lie in its detailed and specific nature. This cross-sectional study in neurointensive care is, to the best of our knowledge, the first of its kind.

## 5. Conclusions

In summary, the implementation of MNT in NICUs exhibits considerable heterogeneity in clinical practice. Substantial disparities between guideline recommendations and their practical application in clinical settings emerged. Our findings indicate a potential lack of feasibility for some MNT strategies and underscore the impact of partially scarce evidence in neurological patients, emphasizing the need for further research and pragmatic recommendations.

## Figures and Tables

**Table 1:** Demographics of participating neurointensivists.

Demographics	Distribution
Age (years), mean (SD)	46.0 (9.2)
Institution	
Academic hospitals, *n* (%)	25/55 (45.5%)
Non-academic hospitals, *n* (%)	30/55 (54.5%)
Subspeciality	
Neurology, *n* (%)	43/55 (78.2%)
Neurosurgery, *n* (%)	5/55 (9.1%)
Anesthesiology, *n* (%)	6/55 (10.9%)
Internal medicine, *n* (%)	1/55 (1.8%)
Others, *n* (%)	0/55 (0.0%)
Structure of NICU	
Neurology led ICU, *n* (%)	21/56 (37.5%)
Neurosurgery led ICU, *n* (%)	5/56 (8.9%)
Interdisciplinary neurology/neurosurgery led ICU, *n* (%)	3/56 (5.4%)
Interdisciplinary anesthesiology/neurology led ICU, *n* (%)	6/56 (10.7%)
Interdisciplinary anesthesiology/neurosurgery led ICU, *n* (%)	4/56 (7.1%)
Interdisciplinary internal medicine/neurology led ICU, *n* (%)	7/56 (12.5%)
interdisciplinary internal medicine/neurosurgery led ICU, *n* (%)	0/56 (0.0%)
Others, *n* (%)	10/56 (17.9%)
Years of medical experience	
0–5 years, *n* (%)	0/55 (0.0%)
5–10 years, *n* (%)	7/55 (12.7%)
>10 years, *n* (%)	48/55 (87.3%)
Years of experience in intensive care medicine	
0–2 years, *n* (%)	4/54 (7.4%)
2–5 years, *n* (%)	14/54 (25.9%)
>5 years, *n* (%)	36/54 (66.7%)
Leadership position on ICU	
Yes, *n* (%)	34/54 (63.0%)
No, *n* (%)	20/54 (37.0%)
Number of intensive care beds on ICU	
0–5, *n* (%)	0/55 (0.0%)
6–10, *n* (%)	17/55 (30.9%)
11–15, *n* (%)	23/55 (41.8%)
>16, *n* (%)	15/55 (27.3%)
Proportionate number of neurological/neurosurgical intensive care beds on ICU	
0–5, *n* (%)	14/54 (25.9%)
6–10, *n* (%)	21/54 (38.9%)
11–15, *n* (%)	9/54 (16.7%)
>16, *n* (%)	0/54 (0.0%)
All, *n* (%)	10/54 (18.5%)
Annual number of neurological/neurosurgical patients on ICU	
0–149, *n* (%)	12/53 (22.6%)
150–299, *n* (%)	10/53 (18.9%)
300–449, *n* (%)	10/53 (18.9%)
450–599, *n* (%)	5/53 (9.4%)
600–750, *n* (%)	1/53 (1.9%)
>750, *n* (%)	7/53 (13.2%)
Unknown, *n* (%)	8/53 (15.1%)

ICU, intensive care unit. *n*, number. SD, standard deviation.

**Table 2:** Guideline adherence.

Survey-Topic	Guideline Adherence	DGEM Strength of Consensus	ESPEN Strength of Consensus	ESPEN Level of Evidence
Existence of SOP/feeding protocol	80% (41/51)	Strong consensus (100%)	Proposed but not specified	-
Implementation of risk stratification at ICU admission	36% (18/50)	Strong consensus (97%)	Strong consensus (100%)	GPP
Use of specific risk stratification scores	20% (10/50)	Consensus (88%)	Not specified	-
Individualized determination of EE	75% (36/48)	Implicit assumption	Implicit assumption	-
Use of indirect calorimetry to determine EE	15% (7/48)	Strong consensus (100%)	Strong consensus (95%)	B
Use of actual body weight to determine EE (non-obese, non-cachectic patients)	49% (18/37)	Strong consensus (94%)	Consensus (89%)	GPP
Hypocaloric energy target in the acute phase of disease (d 0–2)	64% (23/36)	Strong consensus (94%)	Strong consensus (100%)	B
Isocaloric energy target in the post-acute phase (d 3–7)	77% (28/36)	Strong consensus (94%)	Strong consensus (95%)	0
Individualized targets for protein intake	57% (22/39)	Implicit assumption	Implicit assumption	Implicit assumption
Protein target during critical illness 1.0–1.2 g/kgBw/day (DGEM) or ESPEN (1.3 g) in non-obese patients	39% (15/39)	Consensus (88%)	Strong consensus (91%)	0
Protein target 1.5 g (DGEM) or 1.3 g (ESPEN) in obese patients	13% (6/48)	Strong consensus (94%)	Consensus (89%)	GPP
Evaluation of metabolic intolerance	53% (24/45)	Strong consensus (97%)	Proposed but not specified	-
Re-evaluation of EE during critical illness	38% (17/45)	Consensus (89%)	Not specified; note on phases of critical illness	-
**Overall**	**47%**			

**Guideline adherence**: 76–100% adherence (green); >51–75% adherence (yellow); 26–50%adherence (orange); and 0–25% adherence (red). **Strength of consensus**: Strong consensus (>90% of the participants), consensus (>75–90% of the participants), implicit assumption (guideline explicitly speaks about a topic without formulating a recommendation), and proposed but not specified. **ESPEN level of evidence:** B: body of evidence including high-quality systematic reviews of case control or cohort studies, high-quality case control or cohort studies with a very low risk of confounding variables or bias, and a high probability that the relationship is causal. Both directly applicable to the target population; or a body of evidence including well-conducted case control or cohort studies with a low risk of confounding or bias and a moderate probability that the relationship is causal, directly applicable to the target population and demonstrating overall consistency of results; or extrapolated evidence from high quality meta-analyses, systematic reviews of RCTs, or RCTs with a very low risk of bias as well as well-conducted meta-analyses, systematic reviews, or RCTs with a low risk of bias. 0: Evidence level included non-analytic studies (e.g., case reports, case series, and expert opinion); or extrapolated evidence from high-quality systematic reviews of case control or cohort studies, or high-quality case control or cohort studies with a very low risk of confounding variables or bias and high probability that the relationship is causal. This also included well-conducted case control or cohort studies with a low risk of confounding or bias and a moderate probability that the relationship is causal. GPP: good practice points. Recommended best practice based on the clinical experience of the guideline development group. EE, energy expenditure. SOP, standard operating procedure. d, day. g/kgBw/day, grams per kilogram bodyweight per day. (Modified from Singer et al. and Elke et al. [4,6]). Excerpts from the original guidelines can be found in the Supplemental Table S2.

## Data Availability

The data presented in this study are available on request from the corresponding author. The data are not publicly available to protect privacy.

## Supplementary Materials

The following supporting information can be downloaded at: https://www.mdpi.com/article/10.3390/jcm13020447/s1, Table S1. Statistical Analysis of demographic data of our responding NICUs. For comparison the Chi²-test with Yates correction was performed using Graphpad-prism. *p*-value and Chi²-value are displayed in the corresponding columns. Table S2. Original wording of Guideline-adherence. References [4,5,6] are cited in the supplementary materials.

## References

[1] Villet S., Chiolero R.L., Bollmann M.D., Revelly J.P., Cayeux Rn M.C., Delarue J. (2005). Negative impact of hypocaloric feeding and energy balance on clinical outcome in ICU patients. Clin. Nutr..

[2] Ndahimana D., Kim E.K. (2018). Energy Requirements in Critically Ill Patients. Clin. Nutr. Res..

[3] Compher C., Bingham A.L., McCall M., Patel J., Rice T.W., Braunschweig C. (2022). Guidelines for the provision of nutrition support therapy in the adult critically ill patient: The American Society for Parenteral and Enteral Nutrition. J. Parenter. Enter. Nutr..

[4] Singer P., Blaser A.R., Berger M.M., Alhazzani W., Calder P.C., Casaer M.P. (2019). ESPEN guideline on clinical nutrition in the intensive care unit. Clin. Nutr..

[5] Singer P., Blaser A.R., Berger M.M., Calder P.C., Casaer M., Hiesmayr M. (2023). ESPEN practical and partially revised guideline: Clinical nutrition in the intensive care unit. Clin. Nutr..

[6] Elke G., Hartl W.H., Kreymann K.G., Adolph M., Felbinger T.W., Graf T. (2019). Clinical Nutrition in Critical Care Medicine—Guideline of the German Society for Nutritional Medicine (DGEM). Clin. Nutr. ESPEN.

[7] Reintam Blaser A., Starkopf J., Alhazzani W., Berger M.M., Casaer M.P., ESICM Working Group on Gastrointestinal Function (2017). Early enteral nutrition in critically ill patients: ESICM clinical practice guidelines. Intensive Care Med..

[8] Abdelmalik P.A., Dempsey S., Ziai W. (2017). Nutritional and Bioenergetic Considerations in Critically Ill Patients with Acute Neurological Injury. Neurocrit. Care.

[9] Speyer R., Balaguer M., Cugy E., Devoucoux C., Morinière S., Soriano G. (2023). Expert Consensus on Clinical Decision Making in the Disease Trajectory of Oropharyngeal Dysphagia in Adults: An International Delphi Study. J. Clin. Med..

[10] Zuercher P., Schenk N.V., Moret C., Berger D., Abegglen R., Schefold J.C. (2020). Risk Factors for Dysphagia in ICU Patients after Invasive Mechanical Ventilation. Chest.

[11] Olkowski B.F., Shah S.O. (2017). Early Mobilization in the Neuro-ICU: How Far Can We Go?. Neurocrit. Care.

[12] Cotoia A., Umbrello M., Ferrari F., Pota V., Alessandri F., Cortegiani A. (2023). Nutritional support and prevention of post-intensive care syndrome: The Italian SIAARTI survey. J. Anesth. Analg. Crit. Care.

[13] Presser S., Couper M.P., Lessler J.T., Martin E., Martin J., Rothgeb J.M. (2004). Methods For Testing And Evaluating Survey Questions. Public Opin. Q..

[14] Collins D. (2003). Pretesting survey instruments: An overview of cognitive methodso title found. Qual. Life Res..

[15] Martin C.M., Doig G.S., Heyland D.K., Morrison T., Sibbald W.J., Southwestern Ontario Critical Care Research Network (2004). Multicentre, cluster-randomized clinical trial of algorithms for critical-care enteral and parenteral therapy (ACCEPT). CMAJ Can. Med. Assoc. J. J. Assoc. Medicale Can..

[16] Barr J., Hecht M., Flavin K.E., Khorana A., Gould M.K. (2004). Outcomes in Critically Ill Patients Before and After the Implementation of an Evidence-Based Nutritional Management Protocol. Chest.

[17] Mogensen K.M., Robinson M.K., Casey J.D., Gunasekera N.S., Moromizato T., Rawn J.D. (2015). Nutritional Status and Mortality in the Critically Ill*. Crit. Care Med..

[18] Moisey L.L., Mourtzakis M., Cotton B.A., Premji T., Heyland D.K., Wade C.E. (2013). Skeletal muscle predicts ventilator-free days, ICU-free days, and mortality in elderly ICU patients. Crit. Care.

[19] Sabbouh T., Torbey M.T. (2018). Malnutrition in Stroke Patients: Risk Factors, Assessment, and Management. Neurocrit. Care.

[20] Zhang P., Bian Y., Tang Z., Wang F. (2021). Use of Nutrition Risk in Critically Ill (NUTRIC) Scoring System for Nutrition Risk Assessment and Prognosis Prediction in Critically Ill Neurological Patients: A Prospective Observational Study. JPEN J. Parenter. Enter. Nutr..

[21] Chen D., Zhao B., Wang L., Qiu Y., Mao E., Sheng H. (2023). Prognostic performance of the NRS2002, NUTRIC, and modified NUTRIC to identify high nutritional risk in severe acute pancreatitis patients. Front. Nutr..

[22] Tatucu-Babet O.A., Fetterplace K., Lambell K., Miller E., Deane A.M., Ridley E.J. (2020). Is Energy Delivery Guided by Indirect Calorimetry Associated With Improved Clinical Outcomes in Critically Ill Patients? A Systematic Review and Meta-analysis. Nutr. Metab. Insights.

[23] Duan J.Y., Zheng W.H., Zhou H., Xu Y., Huang H.B. (2021). Energy delivery guided by indirect calorimetry in critically ill patients: A systematic review and meta-analysis. Crit. Care.

[24] Singer P., De Waele E., Sanchez C., Ruiz Santana S., Montejo J.C., Laterre P.F. (2021). TICACOS international: A multi-center, randomized, prospective controlled study comparing tight calorie control versus Liberal calorie administration study. Clin. Nutr..

[25] Pertzov B., Bar-Yoseph H., Menndel Y., Bendavid I., Kagan I., Glass Y.D. (2022). The effect of indirect calorimetry guided isocaloric nutrition on mortality in critically ill patients—A systematic review and meta-analysis. Eur. J. Clin. Nutr..

[26] Elke G., Hartl W.H., Adolph M., Angstwurm M., Brunkhorst F.M., Edel A. Laborchemisches und kalorimetrisches Monitoring der medizinischen Ernährungstherapie auf der Intensiv- und Intermediate Care Station: Zweites Positionspapier der Sektion Metabolismus und Ernährung der Deutschen Interdisziplinären Vereinigung für Intensiv- und Notfallmedizin (DIVI). Med Klin—Intensivmed Notfallmedizin [Internet]. 17 April 2023. https://link.springer.com/10.1007/s00063-023-01001-2.

[27] Delsoglio M., Achamrah N., Berger M.M., Pichard C. (2019). Indirect Calorimetry in Clinical Practice. J. Clin. Med..

[28] Zusman O., Kagan I., Bendavid I., Theilla M., Cohen J., Singer P. (2019). Predictive equations versus measured energy expenditure by indirect calorimetry: A retrospective validation. Clin. Nutr..

[29] Wu S., Iqbal S., Giroux M., Alam N., Campisi J., Razek T. (2022). Penn State equation versus indirect calorimetry for nutritional assessment in patients with traumatic brain injury. Can. J. Surg..

[30] Morbitzer K.A., Wilson W.S., Chaben A.C., Darby A., Dehne K.A., Brown E.R. (2019). Energy Expenditure in Critically Ill Adult Patients With Acute Brain Injury: Indirect Calorimetry vs. Predictive Equations. Front. Neurol..

[31] Frankenfield D.C., Ashcraft C.M. (2012). Description and prediction of resting metabolic rate after stroke and traumatic brain injury. Nutrition.

[32] Koukiasa P., Bitzani M., Papaioannou V., Pnevmatikos I. (2015). Resting Energy Expenditure in Critically Ill Patients With Spontaneous Intracranial Hemorrhage. J. Parenter. Enter. Nutr..

[33] McEvoy C.T., Cran G.W., Cooke S.R., Young I.S. (2009). Resting energy expenditure in non-ventilated, non-sedated patients recovering from serious traumatic brain injury: Comparison of prediction equations with indirect calorimetry values. Clin. Nutr..

[34] Heyland D.K., Patel J., Compher C., Rice T.W., Bear D.E., Lee Z.Y. (2023). The effect of higher protein dosing in critically ill patients with high nutritional risk (EFFORT Protein): An international, multicentre, pragmatic, registry-based randomised trial. Lancet.

[35] Hurt R.T., McClave S.A., Martindale R.G., Ochoa Gautier J.B., Coss-Bu J.A., Dickerson R.N. (2017). Summary Points and Consensus Recommendations From the International Protein Summit. Nutr. Clin. Pract. Off Pub. Am. Soc. Parenter. Enter. Nutr..

[36] Weijs P.J., Looijaard W.G., Beishuizen A., Girbes A.R., Oudemans-van Straaten H.M. (2014). Early high protein intake is associated with low mortality and energy overfeeding with high mortality in non-septic mechanically ventilated critically ill patients. Crit. Care.

[37] Deane A.M., Plummer M.P., Ali Abdelhamid Y. (2022). Update on glucose control during and after critical illness. Curr. Opin. Crit. Care.

[38] Sharma S., Hashmi M.F., Castro D. (2023). Hypophosphatemia. StatPearls [Internet].

[39] Bohé J., Abidi H., Brunot V., Klich A., Klouche K., Sedillot N. (2021). Individualised versus conventional glucose control in critically-ill patients: The CONTROLING study-a randomized clinical trial. Intensive Care Med..

[40] Wang W., Xu C., Ma X., Zhang X., Xie P. (2020). Intensive Care Unit-Acquired Weakness: A Review of Recent Progress With a Look Toward the Future. Front. Med..

[41] Watanabe D., Uranaka K., Asazawa K., Akimoto T., Ohnuma H. (2023). Effects of interprofessional conferences on intensive care units: Comparing lengths of stay in the intensive care unit before and after the introduction of interprofessional conferences. J. Rural Med..

[42] Arney B.D., Senter S.A., Schwartz A.C., Meily T., Pelekhaty S. (2019). Effect of Registered Dietitian Nutritionist Order-Writing Privileges on Enteral Nutrition Administration in Selected Intensive Care Units. Nutr. Clin. Pract..

