# Supplementary material for: Survey on Nutrition in Neurological Intensive Care Units (SONNIC)—A Cross-Sectional Survey among German-Speaking Neurointensivists on Medical Nutritional Therapy
Source: J Clin Med. 2024 Jan 13;13(2):447. doi: 10.3390/jcm13020447 (PMC10816503; doi:10.3390/jcm13020447)
Supplement: Supplementary file 1 [file jcm-13-00447-s001.zip › Supplemental Table S1.pdf]

Table S1. Statistical Analysis of demographic data of our responding NICUs
For comparison the Chi^2-test with Yates correction was performed using Graphpad-prism. P-value and Chi^2-value are displayed in the corresponding columns.

	**Does a SOP exist?**	**Is a risk stratification at admission to ICU implemented?**	**Are specific risk stratification scores used?**	**Are patients' caloric needs being determined?**	**Is indirect calorimetry being used?**	**Is the actual body weight being applied in non-obese patients?**	**Is a hypocaloric energy target set in the first 2-3 days?**	**Is an isocaloric energy target set in the following 4-7 days?**	**Is a protein intake target set?**	**Is the protein target during critical illness 1.0-1.2g/kgBw/day (DGEM) or ESPEN (1.3) in non-obese patients?**	**Is the protein target in obese patients either 1.5g (DGEM) or 1.3g (ESPEN)?**	**Is nutritional management in general evaluated?**	**Are patients' caloric needs evaluated?**
**Acadamic Hospital (n1) vs. non-acadamic (n2)**	n1=20/25<br>n2= 21/26<br>p= 0.77<br>Chi^2= 0.08	n1=8/25<br>n2= 9/25<br>p= 0.99<br>Chi^2= 0.01	n1= 5/25<br>n2= 5/25<br>p= 0.99<br>Chi^2= 0.01	n1=19/25<br>n2= 17/23<br>p= 0.87<br>Chi^2= 0.03	n1= 5/25<br>n2= 2/23<br>p= 0.48<br>Chi^2= 0.49	n1= 10/18<br>n2= 7/14<br>p= 0.96<br>Chi^2= 0.01	n1= 11/19<br>n2= 7/17<br>p= 0.50<br>Chi^2= 0.45	n1= 11/19<br>n2= 9/17<br>p= 0.97<br>Chi^2= 0.01	n1= 11/20<br>n2= 11/19<br>p= 0.88<br>Chi^2= 0.02	n1= 7/20<br>n2= 8/19<br>p= 0.90<br>Chi^2= 0.02	n1= 4/25<br>n2= 2/22<br>p= 0.79<br>Chi^2= 0.07	n1= 14/24<br>n2= 10/21<br>p= 0.68<br>Chi^2= 0.18	n1= 9/24<br>n2= 8/21<br>p= 0.79<br>Chi^2= 0.07
**Subspeciality: Neurology (n1) vs. Others (n2)**	n1=30/39<br>n2= 10/11<br>p= 0.55<br>Chi^2= 0.36	n1= 14/38<br>n2= 3/11<br>p= 0.82<br>Chi^2= 0.05	n1= 8/38<br>n2= 2/11<br>p= 0.83<br>Chi^2= 0.05	n1= 25/36<br>n2= 10/11<br>p= 0.30<br>Chi^2= 1.07	n1= 5/36<br>n2= 2/11<br>p= 0.89<br>Chi^2= 0.02	n1= 14/22<br>n2= 3/9<br>p=0.25<br>Chi^2= 1.30	n1=12/25<br>n2= 6/10<br>p= 0.79<br>Chi^2= 0.07	n1= 12/25<br>n2= 7/10<br>p= 0.42<br>Chi^2= 0.65	n1= 17/31<br>n2= 4/7<br>p= 0.76<br>Chi^2= 0.10	n1= 13/31<br>n2= 2/7<br>p= 0.82<br>Chi^2= 0.05	n1= 6/36<br>n2= 0/11<br>p= 0.35<br>Chi^2= 0.87	n1= 19/35<br>n2= 5/10<br>p= 0.90<br>Chi^2= 0.01	n1= 14/35<br>n2= 3/10<br>p= 0.84<br>Chi^2= 0.04
**Leadership position Yes (n1) vs. No (n2)**	n1=27/33<br>n2= 14/18<br>p= 0.98<br>Chi^2=0.01	n1=13/32<br>n2= 4/18<br>p=0.31<br>Chi^2= 1.02	n1= 7/32<br>n2= 3/18<br>p= 0.94<br>Chi^2= 0.01	n1= 24/31<br>n2= 12/17<br>p= 0.86<br>Chi^2= 0.03	n1= 5/31<br>n2= 2/17<br>p= 0.99<br>Chi^2= 0.01	n1= 11/21<br>n2= 7/11<br>p= 0.81<br>Chi^2= 0.06	n1= 13/24<br>n2= 5/12<br>p= 0.724<br>Chi^2= 0.13	n1= 13/24<br>n2= 7/12<br>p= 0.91<br>Chi^2= 0.01	n1= 12/24<br>n2= 10/15<br>p= 0.49<br>Chi^2= 0.48	n1= 10/24<br>n2= 5/15<br>p= 0.86<br>Chi^2= 0.03	n1= 4/31<br>n2= 2/16<br>p= 0.67<br>Chi^2= 0.18	n1= 18/30<br>n2= 6/15<br>p= 0.34<br>Chi^2= 0.90	n1= 14/30<br>n2= 3/15<br>p= 0.16<br>Chi^2= 2.00
**Annul number of patients > 450 (n1) vs. < 450 (n2)**	n1= 12/13<br>n2= 20/28<br>p= 0.27<br>Chi^2= 1.21	n1= 2/13<br>n2= 12/27<br>p=0.14<br>Chi^2= 2.11	n1= 1/13<br>n2= 8/27<br>p= 0.25<br>Chi^2= 1.33	n1= 10/13<br>n2= 19/25<br>p= 0.73<br>Chi^2= 0.12	n1= 3/13<br>n2= 3/25<br>p= 0.67<br>Chi^2= 0.18	n1= 4/9<br>n2= 11/17<br>p= 0.56<br>Chi^2= 0.33	n1= 5/10<br>n2= 11/19<br>p= 0.99<br>Chi^2= 0.01	n1= 7/10<br>n2= 10/17<br>p= 0.87<br>Chi^2= 0.03	n1= 4/11<br>n2= 13/22<br>p= 0.39<br>Chi^2= 0.74	n1= 3/11<br>n2= 9/22<br>p= 0.70<br>Chi^2= 0.15	n1= 0/13<br>n2= 5/25<br>p= 0.29<br>Chi^2= 1.10	n1= 8/13<br>n2= 13/23<br>p= 0.95<br>Chi^2= 0.01	n1= 5/13<br>n2= 9/23<br>p= 0.75<br>Chi^2= 0.10
**Experience in intensive care medicine > 5y (n1) vs.< 5y (n2)**	n1= 28/32<br>n2= 12/18<br>p= 0.16<br>Chi^2= 1.96	n1= 11/31<br>n2= 6/18<br>p=0.87<br>Chi^2= 0.03	n1= 5/31<br>n2= 5/18<br>p= 0.54<br>Chi^2= 0.37	n1= 23/29<br>n2= 12/18<br>p= 0.53<br>Chi^2= 0.39	n1= 6/29<br>n2= 1/18<br>p= 0.32<br>Chi^2= 0.99	n1= 8/19<br>n2= 10/12<br>p= 0.06<br>Chi^2= 3.58	n1= 10/23<br>n2= 8/12<br>p= 0.34<br>Chi^2= 0.90	n1= 13/23<br>n2= 7/12<br>p= 0.80<br>Chi^2= 0.07	n1= 17/26<br>n2= 4/12<br>p= 0.13<br>Chi^2= 2.24	n1= 12/26<br>n2= 3/12<br>p= 0.38<br>Chi^2= 0.78	n1= 2/29<br>n2= 4/18<br>p= 0.28<br>Chi^2= 1.17	n1= 16/28<br>n2= 8/17<br>p= 0.73<br>Chi^2= 0.12	n1= 13/28<br>n2= 4/17<br>p= 0.22<br>Chi^2= 1.49
**Neurology led ICU (n1) vs. Others (n2)**	n1= 17/21<br>n2= 23/29<br>p= 0.83<br>Chi^2= 0.05	n1= 7/21<br>n2= 11/29<br>p= 0.97<br>Chi^2= 0.01	n1= 3/21<br>n2= 7/29<br>p= 0.62<br>Chi^2= 0.25	n1= 15/20<br>n2= 21/28<br>p= 0.74<br>Chi^2= 0.11	n1= 3/20<br>n2= 4/28<br>p= 0.73<br>Chi^2= 0.12	n1= 9/15<br>n2= 9/21<br>p= 0.50<br>Chi^2= 0.46	n1= 5/15<br>n2=13/21<br>p= 0.18<br>Chi^2= 1.83	n1= 6/15<br>n2= 14/21<br>p= 0.21<br>Chi^2= 1.56	n1= 8/17<br>n2= 14/22<br>p= 0.48<br>Chi^2= 0.50	n1= 7/17<br>n2= 8/22<br>p= 0.98<br>Chi^2= 0.01	n1= 2/20<br>n2= 4/28<br>p= 0.99<br>Chi^2= 0.01	n1= 10/20<br>n2= 14/25<br>p= 0.92<br>Chi^2= 0.01	n1= 9/20<br>n2= 8/25<br>p= 0.56<br>Chi^2= 0.34
**MTT (n1) vs. STT (n2)**	n1= 15/19<br>n2= 26/31<br>p= 0.95<br>Chi^2= 0.01	n1= 9/18<br>n2= 9/32<br>p= 0.12<br>Chi^2= 2.39	n1= 5/18<br>n2= 5/32<br>p= 0.51<br>Chi^2= 0.44	n1= 13/17<br>n2= 23/31<br>p= 0.86<br>Chi^2= 0.03	n1= 4/17<br>n2= 3/31<br>p= 0.38<br>Chi^2= 0.76	n1= 6/13<br>n2= 12/23<br>p= 0.99<br>Chi^2= 0.01	n1= 7/13<br>n2= 11/23<br>p= 0.99<br>Chi^2= 0.01	n1= 10/13<br>n2= 10/23<br>p= 0.11<br>Chi^2= 2.53	n1= 6/13<br>n2= 16/26<br>p= 0.57<br>Chi^2= 0.33	n1= 3/13<br>n2= 12/26<br>p= 0.23<br>Chi^2= 1.44	n1= 1/17<br>n2= 5/31<br>p= 0.57<br>Chi^2= 0.33	n1= 10/16<br>n2= 13/28<br>p= 0.48<br>Chi^2= 0.51	n1= 8/16<br>n2= 9/28<br>p= 0.40<br>Chi^2= 0.72
