# Supplementary material for: Survey on Nutrition in Neurological Intensive Care Units (SONNIC)—A Cross-Sectional Survey among German-Speaking Neurointensivists on Medical Nutritional Therapy
Source: J Clin Med. 2024 Jan 13;13(2):447. doi: 10.3390/jcm13020447 (PMC10816503; doi:10.3390/jcm13020447)
Supplement: Supplementary file 1 [file jcm-13-00447-s001.zip › Supplemental Table S2.pdf]

Table S2: Original wording of Guideline-adherence:

Survey-Topic	Guideline-Adherence	Original Wording in DGEM-Guideline (1)	Original Wording in ESPEN-Guideline (2,3)
Existence of SOP/feeding protocol	80% (41/51)	Recommendation 1:<br>"Nutritional therapy should include the use of a feeding protocol".<br>Strong consensus (100%)<br>Page: 225	3.23. Clinical question 24: How should nutrition therapy be monitored during the ICU stay?<br>"We propose standard operating procedures developed in a separate document"<br>Page: 71
Implementation of risk stratification at ICU admission	36% (18/50)	Recommendation 2:<br>"Nutrition status should be assessed at the time of ICU admission".<br>Strong consensus (97%)<br>Page: 226	Recommendation 2:<br>"A general clinical assessment should be performed to assess malnutrition in the ICU, until a specific tool has been validated.<br>Remark: General clinical assessment could include anamnesis, report of unintentional weight loss or decrease in physical performance before ICU admission, physical examination, general assessment of body composition, and muscle mass and strength, if possible".<br>Grade of recommendation: GPP<br>Strong consensus (100% agreement)<br>Page: 53
Use of specific risk stratification scores	20% (10/50)	Recommendation 3a:<br>"At the time of ICU admission, the criteria for disease-specific malnutrition proposed by DGEM, or the subjective global assessment (SGA) may be used to assess nutrition status".<br>Consensus (88%)<br>Page: 227	Recommendation 2:<br>Commentary: Discussion whether scores should be used or not as the authors claim that specific tools have not been validated yet and therefore do not specifically recommend specific risk stratification scores but mention to include them in a general clinical assessment.<br>Page: 53
Individualized determination of EE	75% (36/48)	Implicit assumption, no specific note as a recommendation.	Implicit assumption, no specific note as a recommendation.
Use of indirect calorimetry to determine EE	15% (7/48)	Recommendation 5a:<br>"Indirect calorimetry should be used to determine the energy expenditure/calorie target".<br>Strong consensus (100%)<br>Page: 228	Recommendation 15:<br>"In critically ill mechanically ventilated patients, EE should be determined by using indirect calorimetry".<br>Grade of recommendation: B<br>Strong consensus (95% agreement)<br>Page: 58

Use of actual body weight to determine EE (non-obese, non-cachectic patients)	49% (18/37)	Recommendation 6:<br>“In non-obese patients (BMI <30 kg/m2), the actual body weight may be used for calculating energy expenditure by the estimation formula”.<br>Strong consensus (94%)<br>Page: 229	Recommendation 52 commentary:<br>“If indirect calorimetry is not available (…), the reference (adjusted) body weight should then change from actual body weight to ideal body weight at a BMI > 25 kg/m2”.<br>Grade of recommendation 52: GPP<br>Consensus (89% agreement)<br>Page: 70
Hypocaloric energy target in the acute phase of disease (d 0-2)	64% (23/36)	Recommendation 9a:<br>“Calorie intake should begin with 75% of the measured or estimated energy expenditure (the calorie target) and should be increased subsequently according to individual metabolic tolerance to provide patients with 100% of the calorie target by the end of the acute phase (4-7 days after the onset of critical illness)”.<br>Strong consensus (94%)<br>Page: 229-230	Recommendation 17:<br>“Hypocaloric nutrition (not exceeding 70% of EE) should be administered in the early phase of acute illness”.<br>Grade of recommendation: B<br>Strong consensus (100% agreement)<br>Page: 58
Isocaloric energy target in the post-acute phase (d 3-7)	77% (28/36)	Recommendation 9a:<br>“Calorie intake should begin with 75% of the measured or estimated energy expenditure (the calorie target) and should be increased subsequently according to individual metabolic tolerance to provide patients with 100% of the calorie target by the end of the acute phase (4e7 days after the onset of critical illness)”.<br>Strong consensus (94%)<br>Page: 229-230	Recommendation 18:<br>“After day 3, caloric delivery can be increased up to 80-100% of measured EE”.<br>Grade of recommendation: 0<br>Strong consensus (95% agreement)<br>Page: 58
Individualized targets for protein intake	56% (22/39)	Implicit assumption, no specific note as a recommendation.	Implicit assumption, no specific note as a recommendation.
Protein target during critical illness 1.0-1.2g/kgBw/day (DGEM) or ESPEN (1.3) in non-obese patients	39% (15/39)	Recommendation 14a:<br>“Usually, the target of protein or amino acid intake in the acute phase should be at 1.0 g or 1.2 g/kg of actual body weight per day, respectively”.<br>Consensus (87.5%)<br>Page 235<br><br>Recommendation 15:<br>“In the anabolic recovery phase (convalescence), the protein-/ amino acid intake should be at 100% of the	Recommendation 22:<br>“During critical illness, 1.3 g/kg protein equivalents per day can be delivered progressively”.<br>Grade of recommendation: 0<br>Strong consensus (91% agreement)<br>Page: 61

		target proposed for the acute phase (1.0 g protein or 1.2 g amino acids per kg actual body weight per day)". Consensus (88%) Page: 239	
Protein target 1.5g (DGEM) or 1.3g (ESPEN) in obese patients	13% (6/48)	Recommendation 61: "Usually, in obese critically ill patients (BMI >=30 kg/m^2) the target of protein or amino acid intake in the acute phase should be at 1.5 g protein (or at 1.8 g amino acids)/kg ideal body weight and day". Strong consensus (94%) Page: 262	Recommendation 51: "An iso-caloric high protein diet can be administered to obese patients, preferentially guided by indirect calorimetry measurements and urinary nitrogen losses". Grade of recommendation: 0 Consensus (89% agreement). "If urinary nitrogen losses or lean body mass determination are not available, protein intake can be 1.3 g/kg "adjusted body weight"/d". Grade of recommendation: GPP Consensus (89% agreement) Page: 70
Evaluation of metabolic intolerance	53% (24/45)	Recommendation 9b: "When there are distinct signs of individual metabolic intolerance (blood glucose concentration >180 mg/dL despite an insulin infusion rate >4 IU/h, plasma phosphate concentration <0.65 mmol/L), the calorie/macronutrient intake should be reduced to an extent that tolerance is established again, or that a phosphate supplementation is no longer necessary, respectively (section 6.2.3)". Strong consensus (97%) Page: 230-233	Proposed and spoken about on page 70-72.
Re-evaluation of EE during critical illness	38% (17/45)	Recommendation 4: "MNT should be controlled by the calorie/protein/amino acid intake or corresponding targets recommended for the acute phase, and by individual metabolic tolerance (Recommendations 9aec and 14aec)". Consensus (89%) Page: 227	Not specified, note on phases of critical illness Page: 58
**Overall**	**47%**		

Guideline adherence: 76–100% adherence (green); >51–75% adherence (yellow); 26–50%adherence (orange); and 0–25% adherence (red). Strength of consensus: Strong consensus (>90% of the participants), consensus (>75–90% of the participants), implicit assumption (guideline explicitly speaks about a topic without formulating a recommendation) and proposed but not

specified. ESPEN level of evidence: B: body of evidence including high-quality systematic reviews of case control or cohort studies, high-quality case control or cohort studies with a very low risk of confounding variables or bias, and a high probability that the relationship is causal. Both directly applicable to the target population; or a body of evidence including well-conducted case control or cohort studies with a low risk of confounding or bias and a moderate probability that the relationship is causal, directly applicable to the target population and demonstrating overall consistency of results; or extrapolated evidence from high quality meta-analyses, systematic re-views of RCTs, or RCTs with a very low risk of bias as well as well-conducted meta-analyses, systematic reviews, or RCTs with a low risk of bias. 0: Evidence level included non-analytic studies (e.g., case reports, case series, and expert opinion); or extrapolated evidence from high-quality systematic reviews of case control or cohort studies, or high-quality case control or cohort studies with a very low risk of confounding variables or bias and high probability that the relationship is causal. This also included well-conducted case control or cohort studies with a low risk of con-founding or bias and a moderate probability that the relationship is causal. GPP: good practice points. Recommended best practice based on the clinical experience of the guideline development group. EE, energy expenditure. SOP, standard operating procedure. d, day. g/kgBw/day, grams per kilogram bodyweight per day. (Modified from Singer et al. and Elke et al.) (1–3).

References:

1. Elke G, Hartl WH, Kreymann KG, Adolph M, Felbinger TW, Graf T, u. a. Clinical Nutrition in Critical Care Medicine – Guideline of the German Society for Nutritional Medicine (DGEM). Clin Nutr ESPEN. Oktober 2019;33:220–75.
2. Singer P, Blaser AR, Berger MM, Alhazzani W, Calder PC, Casaer MP, u. a. ESPEN guideline on clinical nutrition in the intensive care unit. Clin Nutr. Februar 2019;38(1):48–79.
3. Singer P, Blaser AR, Berger MM, Calder PC, Casaer M, Hiesmayr M, u. a. ESPEN practical and partially revised guideline: Clinical nutrition in the intensive care unit. Clin Nutr. September 2023;42(9):1671–89.
